# Profile of Stilbenes and Other Phenolics in Fanagoria White and Red Russian Wines

**DOI:** 10.3390/metabo11040231

**Published:** 2021-04-09

**Authors:** Andrey R. Suprun, Alexandra S. Dubrovina, Alexey P. Tyunin, Konstantin V. Kiselev

**Affiliations:** Laboratory of Biotechnology, Federal Scientific Center of the East Asia Terrestrial Biodiversity, FEB RAS, 690022 Vladivostok, Russia; suprun.hi@gmail.com (A.R.S.); dubrovina@biosoil.ru (A.S.D.); tyunin@biosoil.ru (A.P.T.)

**Keywords:** piceatannol, piceid, resveratrol, viniferin

## Abstract

Grapes and wines represent the most important source of edible stilbenes and other phenolic metabolites, which demonstrate a wide range of valuable biological activities. However, there is no information about the profile and content of phenolic compounds in Russian wines. We firstly analyzed phenolics (stilbenes, phenolic acids, and flavonols) in some representatives of Russian wines, including eleven red and seven white Russian wines from Fanagoria, Krasnodarsky Territory. The Russian red wines contained six stilbenes (*trans*-resveratrol, *cis*-resveratrol, *trans*-, *cis*-piceid, *trans*-piceatannol, *δ*-viniferin), while the white wines contained only five stilbenes (*cis*-resveratrol, *trans*-, *cis*-piceid, *trans*-piceatannol, *trans*-resveratrol). More than a half of the total stilbenes in the wines (65% of all stilbenes) were presented by *trans*-piceid and *cis*-piceid, while *trans*-resveratrol reached 16% of all the stilbenes. The red wines also contained six phenolic acids and six flavonols, while the white wines contained six phenolic acids and only three flavonols. Myrecitin-3-O-glucoside, quercetin-3-O-glucoside, and myricetin were the major flavonols in the red wines, while dihydroquercetin-3-O-rhamnoside was the major flavonol in the white wines. The red wines contained markedly higher amounts of stilbenes, phenolic acids, and flavonols than the white wines. Thus, the data showed that young red Russian Fanagoria wines represent a rich source of phenolic compounds. The study also revealed that younger wines were more abundant in phenolics, and wine storage for six months in the dark at +10 °C led to a decrease in the total content of phenolics, primarily monomeric stilbenes and quercetin-3-O-glucoside and quercetin flavonols.

## 1. Introduction

Plants produce a wide range of valuable secondary metabolites, such as alkaloids, terpenoids, natural phenols, etc. Natural phenols, or phenolics, are a class of chemical compounds comprising of phenolic acids, lignins, lignans, anthocyanin, tannins, flavonols, and stilbenes. Plant phenolics play important roles in the plant defense against invading pathogens and their contents are highly induced in response to these and other environmental cues [1,2,3]. The plant-derived stilbenoids attracted special attention, since they are known to exhibit a wide range of beneficial health effects (immunomodulatory, anti-inflammatory, antiangiogenic, cancer chemopreventive, and cardioprotective properties) and show great potential for drug research and development [4,5,6]. The greatest interest of scientists was directed to *trans*-resveratrol (3,5,4′-trihydroxy-*trans*-stilbene or *trans*-resveratrol) and its derivatives, since it possesses valuable biologically active properties [4]. Moreover, *trans*-resveratrol and other monomeric stilbenes (e.g., pinosylvin or piceatannol) are important precursors in the biosynthesis of other stilbenes [3]. Plant stilbenes are formed via the phenylpropanoid pathway in plants where stilbene synthase (STS; EC 2.3.1.95) catalyzes the formation of monomeric stilbene backbone condensing three malonyl-CoA molecules and the CoA-ester of a cinnamic acid derivative [7]. Then, the monomeric stilbenes may undergo different types of modifications, including oligomerization, glycosylation, methoxylation, or prenylation, and give rise to other stilbenoids [8,9,10].

Other phenolic compounds are also known for their valuable biologically active properties and health-promoting effects. For example, flavonols are bioactive compounds found in a variety of vegetables and fruits, and their anti-cancer and anti-microbial effects are well known [11,12,13]. Additionally, flavonol aglycones in plants are potent antioxidants that serve to protect the plant from reactive oxygen species, ultraviolet radiation, and form flower color [14,15].

While stilbenes are found in many plant species, grapes and wines produce especially high diversity and amounts of stilbenes [3,16]. The grape is a fruit that is eaten raw or in juice, although it is chiefly used for making wine, and the content of stilbenes in wines is usually considerably higher than in other sources [17,18]. For example, the *trans*-resveratrol content reached 5.3 mg/L in some red Brazilian wines, whereas the total stilbene content reached 87.5 mg/L (Table 1). A higher stilbene content is a feature of red wines, while their content in white varieties is much lower (Table 1). Therefore, it can be assumed that people get the main amount of plant stilbenes via wine consumption. Therefore, studying the contents of stilbenes and other phenolics in red and white wines is important for wine production of high nutritional value. To the best of our knowledge, there is no information on the profile and content of phenolic compounds in Russian wines. Using high-performance liquid chromatography with high-resolution mass spectrometry (HPLC-HRMS), we first analyzed the profile and content of phenolics (stilbenes, phenolic acids, and flavonols) in eleven red and seven white Russian wine varieties (Fanagoria, Krasnodarsky Territory, Russia).

## 2. Results and Discussion

### 2.1. Phenolic Levels in White and Red Fanagoria Wines

In this study, we analyzed 7 white and 11 red wines from the Fanagoria Winery (Krasnodarsky Territory, Russia) for the composition and content of stilbenes, phenolic acids, and flavonols. All white wines contained low amounts of stilbenes, being detected from 0.07 to 1.60 mg/L. Only one white wine sample contained more than 1 mg/L of stilbenes, namely Fanagoria Riesling (Table 2). In white wines, we detected only five stilbenes (*cis*-resveratrol, *trans*-piceid, *cis*-piceid, and *trans*-piceatannol). *δ*-viniferin and *trans*-resveratrol were not detected or found in trace amounts (Table 2).

However, all red wines contained considerably higher amounts of stilbenes than white wines showing 5.74–37.20 mg/L of stilbenes (Table 3), which was 23–82 times higher in comparison with stilbene concentrations in white wines (Table 2).

The highest stilbene levels were detected in the Fanagoria Merlot, Fanagoria Merlot semi-sweet, and Fanagoria Tsimlyansky black wines (Table 3). The lowest stilbene concentration among red wines was detected in the Fanagoria Saperavi semi-sweet, Fanagoria Author’s #1, and Fanagoria Saperavi wines. Russian red wines contained six stilbenes: *trans*-resveratrol, *cis*-resveratrol, *trans*-piceid, *cis*-piceid, *trans*-piceatannol, and *δ*-viniferin. More than a half of the total stilbenes in the red wines (~65% of all stilbenes) were presented by *trans*-piceid and *cis*-piceid. Importantly, *trans*-resveratrol reached a high portion of all the stilbenic compounds (0.7–3.9 mg/L or ~16% of all stilbenes). Other stilbenes were present in lower amounts, less than 20% in total.

The content of *trans*-resveratrol in red wines correlated (R = 0.93) with the total content of stilbenes and reached the highest concentrations in Fanagoria Merlot, Fanagoria Merlot semi-sweet, Fanagoria Cabernet-Saperavi, and Fanagoria Tsimlyansky black varieties (Table 3), being 4.98, 4.56, 3.93, and 2.79 mg/L, respectively. On average, Fanagoria red wines contained 2.7 mg/L of *trans*-resveratrol (Table 3).

**Figure 1:**
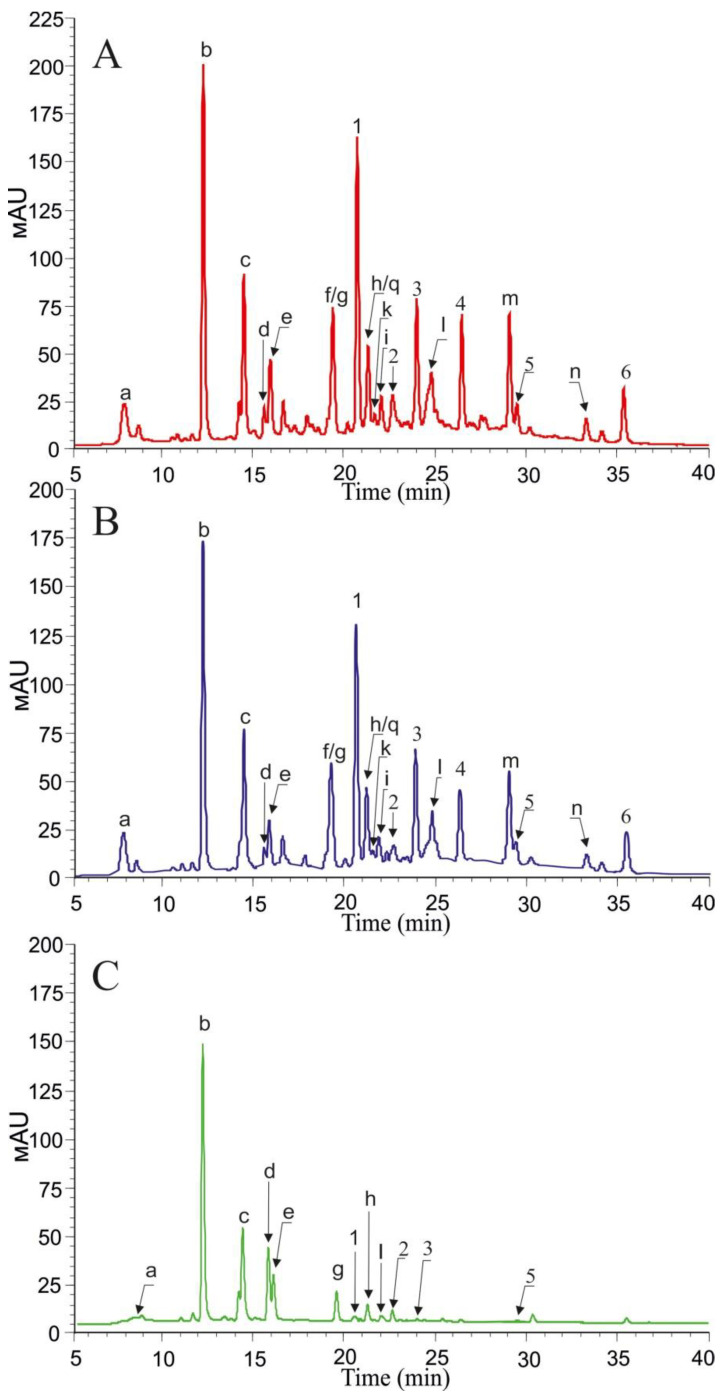
A representative HPLC-UV profile (310 nm) of the extracts from red wine (Fanagoria Merlot, NR collection, vintage 2016), analyzed immediately after opening (**A**) and after six months of storage in the dark at +10 °C (**B**); HPLC-UV profile (310 nm) of the extracts from white wine (Fanagoria Riesling, Fine Select, vintage 2017), analyzed immediately after opening (**C**). *Trans*-piceid (1), *trans*-piceatannol (2), *cis*-piceid (3), *trans*-resveratrol (4), *cis*-resveratrol (5), *δ*-viniferin (6). (a) gallic acid, (b) caftaric acid, (c) coutaric acid, (d) caffeic acid, (e) fertaric acid, (f) myrecitin-3-O-glucoside, (g) *p*-coumaric acid, (h) hexose ester of protocatechuic acid, (i) dihydroquercetin-3-O-rhamnoside, (q) quercetin-3-O-glucoside, (k) quercetin-3-O-glucuronide, (l) myricetin, (m) quercetin, (n) kaempferol.

According to Figure 1, there were other peaks in the wine extract samples, many of which were larger than the stilbene peaks. Using HPLC-MS, we detected the presence of 14 other phenolic compounds (Figure 1, Tables S2–S4), including gallic acid (a, hy-droxybenzoic acid), caffeic and *p*-coumaric acids (d,g, hyroxycinnamic acids), caftaric, coutaric, and fertaric acids (b,c,e, hydroxycinnamoyltartaric acids), myrecitin-3-O-glucoside, quercetin-3-O-glucoside, quercetin-3-O-glucuronide, dihydroquercetin-3-O-rhamnoside, myricetin, quercetin, kaempferol (f,q,k,i,l,m,n, flavonols), and hexose ester of protocatechuic acid (h, flavan-3-ols). The content of these substances reached 42 mg/L in white wines (Fanagoria Muscat semi-sweet, 2017) and 250 mg/L in red wines (Fanagoria Authors’s #1, 2017). In addition to stilbenes, white wines mainly contained phenolic acids, which are the precursors of other high molecular weight phenolics. Red wines contained both the phenolic acids and flavonols, including myrecitin-3-O-glucoside (36–79 mg/L), quercetin-3-O-glucoside (9–30.5 mg/L), quercetin-3-O-glucuronide (2.3–5.9 mg/L), myricetin (6.5–27.7 mg/L), quercetin (2.7–15.2 mg/L), and kaempferol (0.8–4.4 mg/L). The total content of flavonols in red wines reached 120 mg/L (Table S3). Notably, approximately the same amount of flavonols was detected in red wines of two different Spain grape varieties, which were Tempranillo and Graciano [23]. Moreover, quercetin composition in red Russian wine (7.8 ± 0.9 mg/L) was similar for both Canadian and American Merlots (8.4 ± 0.7 and 8.5 ± 0.8 mg/L, respectively), but was lower than in Chilean wines (14.1 ± 0.9) [24].

Interestingly, we noted that 10–100 mg of *trans*-resveratrol per kg of animal weight was used in animal studies at every experimental day [25,26]. According to Liu et al. [25], or Cheng et al. [26], 650 mg of resveratrol must be consumed daily in the human diet (10 mg of *trans*-resveratrol per 65 kg of the average human weight) to reach the lower limits of the therapeutic doses of *trans*-resveratrol used in animal experiments. Consumption of wines with the highest content of *trans*-resveratrol (Fanagoria Merlot, F-Style, 2017, *trans*-resveratrol content is 5 mg/L) would require drinking 130 L (650 mg/5 mg/L = 130 L) to provide the therapeutic dose used in the animal experiments, which is not physically possible. Flavonols are bioactive compounds found in a variety of vegetables and fruits, and its anti-cancer and anti-microbial effects are well known [16,17,18]. In total, our wines contained up to 37 mg of stilbenes and up to 120 mg of flavonoids, which means that the content of these substances reached 150 mg/L, which would require drinking ~4 L of wine, which is also a large volume. One could suppose that there might be more stilbenes in the grape juices, but data reported previously [18] demonstrates that the available commercial juices contained much less stilbene amounts than red wines (0.2–2.3 mg/L). Therefore, the grape juices cannot be used as a stilbene source to provide the therapeutic dose used in the experiments.

Goldberg et al. [27] concluded that the amount of resveratrol absorbed by drinkers of red wine is small enough that it is unlikely to explain the French paradox, a phenomenon denoting that French people have relatively low incidences of coronary heart disease, while having a diet relatively rich in saturated fats. However, if we take into account all the phenolic compounds with potential anti-cancer and other valuable properties that are present in wine, then their total amount is close to low therapeutic doses. Therefore, it is important to count the sum of all phenolic compounds with potential beneficial properties, and not just resveratrol, in wines. It is possible that if we consider the health-beneficial effects of wine-derived stilbenes and other phenolics, it will be necessary to assume much lower doses of resveratrol than those reported in current experiments with animals and to observe the long-term effects of phenolic consumption. At present, such studies are not available in the literature.

### 2.2. Phenolic Levels in Red Wines of Different Vintages and after Storage for Six Months

We compared the content of stilbenes in four wine varieties with a high content of stilbenes, including Fanagoria Cahors Canonical, Fanagoria Merlot, Fanagoria Saperavi semi-sweet, and Fanagoria Tsimlyansky black obtained from the harvest of 2016 and 2017 (Table 3).

In the two wine varieties, the stilbene content did not significantly differ between the two vintages (Fanagoria Merlot and Fanagoria Saperavi semi-sweet). In the other two wine varieties (Fanagoria Cahors Canonical and Fanagoria Tsimlyansky black), stilbene content was significantly lower in the harvest of 2016 than that in the varieties of 2017 harvest (Table 3). The difference in stilbene content between the vintages could result from different amounts of stilbenes in the original plant material or different technological processes (e.g., length of maceration, temperature). In addition, stilbene content could decrease over time during storage. To answer this question, we analyzed the Fanagoria Merlot wine samples before and after storage for six months (in the dark at +10 °C).

As shown in the Table 3 and Figure 1, the storage for six months led to a decrease in the total content of stilbenes by 1.2 times, primarily due to a significant decrease in the content of *trans*-piceatannol, *trans*-resveratrol, *cis*-resveratrol, and *δ*-viniferin (Table 3). The content of *trans*-resveratrol after the storage decreased from 14.1% to 12.4% of total detected stilbenes. Our results also showed a decrease in the content of phenolic acids and flavonols during wine storage (Table S3), except for myricetin and quercetin-3-O-glucuronide content, the amount of which, on the contrary, significantly increased by 1.2-and 2.1-fold, respectively (Table S3).

Similarly, a decrease in the content of bioactive compounds was detected for anthocyanins in the bog bilberry syrup wine after six months of storage [28]. Unfortunately, we did not find any other examples of similar studies on wine storage effects in the literature.

## 3. Materials and Methods

### 3.1. Wine Samples

We analyzed 18 (11 red and 7 white wines) samples of wine of various varieties: Author’s #1, Cabernet, Cabernet-Saperavi, Cahors Canonical, Chardonnay, Merlot F-Style, Merlot NR, Merlot semi-sweet, Muscat semi-sweet, Riesling Fine Select, Riesling NR, Saperavi, Saperavi semi-sweet, Sauvignon, Shardone semi-sweet white, Red semi-sweet, Tsimlyansky black, and White semi-sweet from Fanagoria winery (Krasnodarsky Territory, Russia). In our work, we investigated the main mass-produced Fanagoria wines available for wide sales.

All listed wines were of the 2017 vintage. In addition, the wines with the highest phenolics content were of the 2016 vintage (Fanagoria Cahors Canonical, Fanagoria Merlot, Fanagoria Saperavi semi-sweet, Fanagoria Tsimlyansky black). Moreover, we decided to analyze the changes in the content of phenolic compounds during 6 months of wine storage (in the dark at +10 °C) for wines with a high phenolics content (Fanagoria Merlot, NR collection, vintage 2016). Thus, we analyzed 23 wine samples in three independent repetitions (probes from different bottles).

The vineyards of the company Fanagoria are located in the Temryuksky district of the Krasnodar Territory (Taman Peninsula). This area has a subtropical climate, with warm winters (2.4–3.4 °C average temperature) and hot summers (21.8–25.1 °C average temperature). The amount of precipitation is 53–94 mm per month, and 822 mm per year.

The wine samples of the 2016 vintage were bottled in 2017, and the wine samples of the 2017 vintage were bottled in 2018 (Table S1). We analyzed all the wines in the first half of 2019, which means that, on average, six months or a year have passed after bottling (wine storage on the shelves of the store—10–15 °C, semidarkness). All analyzed wines were obtained in Russia (Vladivostok).

### 3.2. Analytical Chromatography and Mass-Spectrometry (MS)

5 mL of wine or juice sample was strongly mixed for 5 min with 5 mL of the ethyl acetate and after centrifugated at 4000 rpm (PE-6900, Ekroskhim, Russia) for 2 min. After centrifugation, we transferred 5 mL of the ethyl acetate upper fraction by 1 mL to 5 separate 1.5 mL tubes and dried it in a rotary concentrator for 2 h at 35 °C (Concentrator plus, Eppendorf, Germany). After drying, we dissolved the precipitate in each tube in 0.2 mL of ethyl alcohol, and after, we combined the extract from all test tubes and purified it with Discovery^®^ DSC-18 SPE Tube bed wt. 50 mg, volume 1 mL (Supelco, Bellefonte, PA, USA) and then used for HPLC analysis. The measurement for each wine sample was repeated 3 times, and samples were taken from different bottles.

Identification and quantification of all components was achieved by comparison with the commercially available standards and by MS detection. The targeted HPLC with high-resolution mass spectrometry (HPLC-HRMS) of all components was performed using a HPLC LC-20AD XR analytical system (Shimadzu, Japan). The HPLC system was connected with a LCMS-IT-TOF tandem mass-spectrometer (Shimadzu, Kyoto, Japan) [29,30].

The chromatographic separation was performed on a Shim-pack GIST C18 column (150 mm, 2.1-nm i.d., 3-µm part size, Shimadzu, Japan); the column temperature was 40 °C. The mobile phase consisted of A (0.1% aqueous acetic acid) and B (0.1% acetic acid in acetonitrile), which was maintained at a constant flow rate of 0.20 mL/min. The gradient program was used as follows: 0 min 0% of B; 35 min 40% of B; 40 min 50% of B; 50 min 100% of B and then eluent B until 65 min. The injected volume was 2 µL. Compounds were detected in negative ion mode at electrospray ionization (ESI) conditions. The ion source temperature was 210 °C, the range of detection was *m*/*z* 100–1000, and the potential in the ion source was −4 kV. The drying gas (N2) pressure was 100 kPa. The nebulizer gas (N2) flow rate was 1.5 L/min. MS data were collected and processed using the Shimadzu LCMS Solution control and processing software (v.361). UV spectra were registered in the 200–600 nm range, and chromatograms for quantification were used at 310 nm [31]. The contents of component were determined by using external standard method using the five-point regression calibration curves built with the available standards.

All solvents were of HPLC grade. The analytical standards: *trans*-resveratrol, *trans*-piceid, *trans*-piceatannol, caffeic acid, *p*-coumaric acid, kaempferol, myricetin, quercetin, caftaric acid, *trans*-coutaric acid and *trans*-fertaric acid were obtained from Sigma-Aldrich (St. Louis, MO, USA), *δ*-viniferin was obtained from Panreac AppliChem (GmbH, Darmstadt, Germany), gallic acid hydrate from TCI (Tokyo, Japan). Cis isomers of resveratrol and piceid were obtained under sunlight exposure of the respective standard solution containing the *trans*-isomer as reported earlier [32,33].

### 3.3. Data Analysis

Comparisons among groups were made using one-way analysis of variance (ANOVA) followed by Tukey’s test for multiple comparisons. The results were considered statistically significant between groups with *p* values < 0.05.

## 4. Conclusions

This study firstly described the profile and content of stilbenes and other phenolics in Russian wines (winery Fanogoria). The analyzed Russian red wines exhibited one of the highest known *trans*-resveratrol contents (especially Fanagoria Merlot, Fanagoria Merlot semi-sweet, and Fanagoria Tsimlyansky black varieties). At the same time, the content of *trans*-resveratrol in the analyzed Russian white wines was one of the lowest as compared to the white wine varieties collected in other countries.

The data obtained confirmed previously obtained results for other wines indicating that red wines contain higher levels of stilbenes, especially *trans*-resveratrol, phenolic acids and flavonols, than white wines. The Fanagoria wine varieties were identified as wines with a high content of phenolics and representing a rich source of these compounds as other renowned world wines.

## Figures and Tables

**Table 1:** The content of stilbenes in wines of different origins (mg/L).

Wines	*trans*-resveratrol	*cis*-resveratrol	*trans*-piceid	*cis*-piceid	Total Stilbenes	Reference
Red wines						
Brazilian wines	0.0–5.34	1.7–23.23	0.0–20.0	-	2.55–87.52	[19]
French wines	0.9–3.8	0.0–0.9	0.1–26.0	0.0–24.1	-	[19]
Japanese wines	0.13–2.25	0.0–2.66	0.17–3.54	0.37–6.56	0.82–13.43	[20]
Italian wines	0.58–1.34	0.38–0.89	-	-	-	[21]
Russian wines	0.87–4.98	0.0–2.97	1.99–17.78	1.35–10.23	5.74–37.20	present work
Serbian wines	0.11–1.69	0.12–1.49	-	-	-	[22]
White and rose wines						
French wines	0.0–0.2	0.0–0.1	0.0–2.9	0.0–0.9	-	[19]
Japanese wines	0.01–0.66	0.0–0.19	0.02–0.75	0.04–1.50	0.12–3.02	[20]
Russian wines	0.0–0.003	0.0–0.07	0.02–0.70	0.0–0.29	0.18–1.60	present work
Serbian wines	0.02–0.34	0.05–0.58	-	-	-	[22]

**Table 2:** The content of stilbenes in the white Russian Fanogoria wines (mg/L).

White Wine and Vintage	Wine Collection	Wine Classification	*trans*-piceid	*trans*-piceatannol	*cis*-piceid	*trans*-resveratrol	*cis*-resveratrol	*δ*-viniferin	Total
Fanagoria Chardonnay, 2017	NR	dry	0.105 ± 0.005 ^c^	0 ^d^	0 ^b^	0 ^a^	0.072 ± 0.038 ^a^	0 ^a^	0.177 ± 0.053 ^c,d^
Fanagoria Riesling, 2017	NR	semi-dry	0.250 ± 0.069 ^b^	0.361 ± 0.012 ^a,b^	0.211 ± 0.088 ^a^	0 ^a^	0 ^c^	0 ^a^	0.821 ± 0.162 ^b^
Fanagoria Riesling, 2017	Fine Select	semi-dry	0.704 ± 0.194 ^a^	0.565 ± 0.115 ^a^	0.285 ± 0.045 ^a^	0 ^a^	0.048 ± 0.008 ^a^	0 ^a^	1.601 ± 0.335 ^a^
Fanagoria Sauvignon, 2017	NR	dry	0.016 ± 0.005 ^e^	0 ^d^	0.053 ± 0.010 ^b^	0 ^a^	0 ^c^	0 ^a^	0.068 ± 0.005 ^e^
Fanagoria Shardone semi-sweet white, 2017	Fine Select	semi-sweet	0.075 ± 0.045 ^c,d^	0 ^d^	0 ^b^	0.003 ± 0.002 ^a^	0.045 ± 0.025 ^a,b^	0 ^a^	0.123 ± 0.052 ^d^
Fanagoria Muscat semi-sweet, 2017	1957	semi-sweet	0.275 ± 0.085 ^b^	0.031 ± 0.009 ^c^	0 ^b^	0 ^a^	0.019 ± 0.002 ^b^	0 ^a^	0.604 ± 0.175 ^b^
Fanagoria White semi-sweet, 2017	1957	semi-sweet	0.056 ± 0.005 ^d^	0.149 ± 0.011 ^b^	0 ^b^	0 ^a^	0.015 ± 0.002 ^b^	0 ^a^	0.220 ± 0.016 ^c^
Average level			0.185	0.173	0.068	0.001	0.025	0	0.452

^a–d^ Means followed by the same letter were not different using Student’s *t*-test; NR—Nomernoy Reserve or Licence Stockpile. The measurement for each wine sample was repeated 3 times, samples were taken from different bottles. *p* < 0.05 was considered statistically significant.

**Table 3:** The content of stilbenes in the red Russian Fanogoria wines (mg/L).

Red Wine and Vintage	Wine Collection	Wine Classification	Content, mg/L						
			*trans*-piceid	*trans*-piceatannol	*cis*-piceid	*trans*-resveratrol	*cis*-resveratrol	*δ*-viniferin	Total
Fanagoria Author’s #1, 2017	author’s collection	dry	2.565 ± 0.172 ^h^	1.719 ± 0.177 ^b^	1.625 ± 0.245 ^g^	0.865 ± 0.148 ^e,f^	0 ^g^	0.071 ± 0.014 ^e^	6.845 ± 0.425 ^g^
Fanagoria Cabernet, 2017	Licence stockpile	dry	4.025 ± 0.325 ^f^	0^f^	2.536 ± 0.093 ^f^	1.665 ± 0.364 ^d,e^	1.041 ± 0.106 ^c^	1.348 ± 0.045 ^a^	10.615 ± 1.192 ^e^
Fanagoria Cabernet-Saperavi, 2017	author’s collection	dry	5.624 ± 0.453 ^e^	0^f^	3.211 ± 0.063 ^e^	3.930 ± 0.156 ^b^	1.714 ± 0.244 ^a,b^	0 ^f^	14.479 ± 0.311 ^d^
Fanagoria Cahors Canonical, 2016	1957	sweet	2.651 ± 0.603 ^c,d^	0.149 ± 0.039 ^e^	2.136 ± 0.265 ^f^	0.645 ± 0.138 ^f^	0.529 ± 0.071 ^e^	0.150 ± 0.027 ^d^	6.260 ± 1.123 ^g^
Fanagoria Cahors Canonical, 2017	1957	sweet	4.045± 0.201 ^f^	0.676 ± 0.015 ^d^	3.190 ± 0.279 ^e^	1.594 ± 0.103 ^d^	0.726 ± 0.128 ^d^	1.305 ± 0.134 ^a^	11.536 ± 0.433 ^e^
Fanagoria Merlot semi-sweet, 2017	Fine Select	semi-sweet	16.547 ± 0.344 ^a,b^	1.165 ± 0.133 ^b,c^	7.761 ± 0.079 ^b^	4.562 ± 0.117 ^a^	1.065 ± 0.215 ^c^	0.584± 0.018 ^b,c^	31.684 ± 0.225 ^b^
Fanagoria Merlot, 2016	NR	dry	15.072 ± 0.626 ^b,c^	0.906 ± 0.047 ^c^	7.690 ± 0.221 ^b^	4.234 ± 0.135 ^a,b^	1.493 ± 0.017 ^b^	0.573 ± 0.074 ^b,c^	29.968 ± 1.174 ^b^
Fanagoria Merlot, 2016, after six months, storage in dark, +10 °C	NR	dry	13.202 ± 1.186 ^c,d^	0.655 ± 0.072 ^d^	6.329 ± 0.713 ^c^	2.993 ± 0.344 ^c^	0.933 ± 0.05 ^c^	0.051 ± 0.018 ^e^	24.163 ± 1.715 ^c^
Fanagoria Merlot, 2017	NR	dry	10.885 ± 1.124 ^d^	1.760 ± 0.292 ^b^	6.163 ± 0.830 ^c^	4.368 ± 0.129 ^a^	1.467 ± 0.059 ^b^	0.525 ± 0.064 ^c^	25.168 ± 1.819 ^c^
Fanagoria Merlot, 2017	F-Style	dry	17.775 ± 0.575 ^a^	1.470 ± 0.456 ^b^	10.231 ± 0.378 ^a^	4.984 ± 0.275 ^a^	1.972 ± 0.033 ^a^	0.765 ± 0.134 ^b^	37.197 ± 1.598 ^a^
Fanagoria Red semi-sweet, 2017	1957	semi-sweet	4.285 ± 0.176 ^f^	0.953± 0.192 ^c^	2.124 ± 0.075 ^f^	2.482 ± 0.098 ^c^	0.971 ± 0.019 ^c,d^	0.580 ± 0.036 ^b,c^	11.395 ± 0.243 ^e^
Fanagoria Saperavi, 2017	1957	dry	3.379 ± 0.116 ^g^	0.592 ± 0.068 ^d^	1.351 ± 0.805 ^g^	1.246 ± 0.108 ^d,e^	0.805 ± 0.072 ^c^	0.055 ± 0.006 ^e^	7.428 ± 0.865 ^f^
Fanagoria Saperavi semi-sweet, 2016	1957	semi-sweet	2.485 ± 0.162 ^h^	0.896 ± 0.044 ^c^	2.478 ± 0.110 ^f^	0.854 ± 0.037 ^d,e^	0.368 ± 0.019 ^f^	0.059 ± 0.009 ^e^	7.140 ± 0.382 ^f^
Fanagoria Saperavi semi-sweet, 2017	1957	semi-sweet	1.990 ± 0.259 ^e^	0.606 ± 0.177 ^d^	1.684 ± 0.354 ^g^	1.005 ± 0.171 ^e,f^	0.298 ± 0.071 ^f^	0.153 ± 0.052 ^d^	5.736 ± 0.904 ^g^
Fanagoria Tsimlyansky black, 2016	NR	dry	4.054 ± 0.051 ^f^	2.070 ± 0.027 ^b^	2.867 ± 0.052 ^f^	1.908 ± 0.022 ^d^	0.949 ± 0.047 ^c^	0.848 ± 0.113 ^b,c^	12.696 ± 0.315 ^e^
Fanagoria Tsimlyansky black, 2017	NR	dry	3.909 ± 0.124 ^f^	4.073 ± 0.185 ^a^	3.927 ± 0.153 ^d^	2.794 ± 0.067 ^c^	1.145 ± 0.046 ^b,c^	0.602 ± 0.098 ^b,c^	16.450 ± 0.211 ^d^
Average level			6.821	1.183	3.981	2.682	1.018	0.544	16.229

^a–g^ Means followed by the same letter were not different using Student’s *t*-test; NR—Nomernoy Reserve or Licence Stockpile. The measurement for each wine cultivated variety was repeated 3 times, samples were taken from different bottles. *p* < 0.05 was considered statistically significant.

## Data Availability

The data presented in this study are available on request from the corresponding author.

## Supplementary Materials

The following are available online at https://www.mdpi.com/article/10.3390/metabo11040231/s1, Table S1: The bottling date, nutritional value, alcohol, carbohydrates, sugars in the white and red Russian Fanogoria wines, Table S2. The content of phenolic acids and flavonols in the white Russian Fanogoria wines (mg/L), Table S3. The content of phenolic acids and flavonols in the red Russian Fanogoria wines (mg/L), Table S4. List of the phenolic compounds from wine extracts.
