# Supplementary material for: Profile of Stilbenes and Other Phenolics in Fanagoria White and Red Russian Wines
Source: Metabolites. 2021 Apr 9;11(4):231. doi: 10.3390/metabo11040231 (PMC8070200; doi:10.3390/metabo11040231)
Supplement: Supplementary file 1 [file metabolites-11-00231-s001.pdf]

Supplementary matherial for Suprun et al. “Stilbene and other phenolic compounds profile in Russian Fanagoria white and red wines”, Metabolites, 2021.

Table S1. The bottling date, nutritional value, alcohol, carbohydrates, sugars in the white and red Russian Fanogoria wines.

White wine and vintage	Bottling date	Nutritional value (100 ml, kJ)	Alcohol, %	Carbohydrates per 100 ml/g	Sugars, g/l
Fanagoria CHARDONNAY, 2017	22.09.2018	390	12-14	n.i.	no
Fanagoria Riesling, 2017 (NR)	11.10.2018	310	12	1.3	13
Fanagoria Riesling, 2017 (FS)	08.10.2018	410	11-13	3.5	25-35
Fanagoria Sauvignon, 2017	08.10.2018	390	14	n.i.	no
Fanagoria Shardone semi-sweet white, 2017	29.06.2018	410	11-13	3.5	25-35
Fanagoria Muscat semi-sweet, 2017	07.11.2018	340	11	3.5	25-35
Fanagoria White semi-sweet, 2017	10.09.2018	410	11-13	3.5	25-35
Red wine and vintage					
Fanagoria Author's #1, 2017	23.03.2018	410	13.5	n.i.	no
Fanagoria Cabernet, 2017	19.09.2018	320	12-14	n.i.	no
Fanagoria Cabernet-Saperavi, 2017	22.09.2018	320	13	n.i.	no
Fanagoria Cahors Canonical, 2017	19.10.2018	400	10.5	5	50
Fanagoria Merlot semi-sweet, 2017	27.11.2018	320	12-14	3.5	25-35
Fanagoria Merlot (NR), 2017	02.10.2018	340	11-13	n.i.	no
Fanagoria Merlot (FS), 2017	25.10.2018	340	13	n.i.	no
Fanagoria Red semi-sweet, 2017	18.09.2018	340	11-13	3.5	25-35
Fanagoria Saperavi, 2017	27.08.2018	390	12-14	n.i.	no
Fanagoria Saperavi semi-sweet, 2017	16.10.2018	340	11-13	3.5	25-35
Fanagoria Tsimlyansky black, 2017	13.09.2018	380	12.5	n.i.	no

n.i. – no information. The information was taken from the wine labels provided by the manufacturer.

Table S2. The content of phenolic acids and flavonols in the white Russian Fanogoria wines (mg/L).

White wine and vintage	a	b	c	d	e	f	g	h	i	q	k	l	m	n
Fanagoria CHARDONNAY, 2017	1.451 ±0.082^{a}	3.892 ±0.177^{d}	3.310 ±0.185^{cd}	0.519 ±0.080^{c}	4.985 ±0.215^{b}	0^{a}	3.320 ±0.156^{a}	2.011 ±0.177^{a}	0.253 ±0.037^{e}	0^{a}	0^{a}	0^{a}	0^{b}	0^{a}
Fanagoria Riesling, 2017 (NR)	0^{c}	18.745 ±0.605^{a}	6.110 ±0.243^{b}	2.511 ±0.116^{a}	3.502 ±0.153^{c}	0^{a}	1.005 ±0.075^{d}	1.252 ±0.122^{b}	1.791 ±0.116^{b}	0^{a}	0^{a}	0^{a}	0^{b}	0^{a}
Fanagoria Riesling, 2017 (FS)	0^{c}	20.645 ±0.805^{a}	6.335 ±0.505^{b}	2.888 ±0.093^{a}	6.970 ±0.89^{a}	0^{a}	2.242 ±0.384^{b}	1.970 ±0.326^{ab}	2.825 ±0.585^{a}	0^{a}	0^{a}	0^{a}	0^{b}	0^{a}
Fanagoria Sauvignon, 2017	1.185 ±0.035^{a}	1.585 ±0.055	1.758 ±0.243^{e}	0.395 ±0.015^{c}	1.705 ±0.015^{d}	0^{a}	0.935 ±0.035^{d}	0.815 ±0.025^{c}	0.122 ±0.017^{f}	0^{a}	0^{a}	0^{a}	0^{b}	0^{a}
Fanagoria Shardone semi-sweet white, 2017	0^{c}	4.891 ±0.165^{c}	2.590 ±0.091^{de}	0.625 ±0.035^{c}	2.831 ±0.354^{cd}	0^{a}	2.185 ±0.225^{b}	0.857 ±0.130^{c}	0.435 ±0.035^{d}	0^{a}	0^{a}	0^{a}	0^{b}	0^{a}
Fanagoria Muscat semi-sweet, 2017	0^{c}	21.335 ±1.255^{a}	10.575 ±0.775^{a}	1.288 ±0.114^{b}	3.525 ±0.135^{c}	0^{a}	1.935 ±0.025^{c}	0.845 ±0.065^{c}	1.785 ±0.225^{b}	0^{a}	0^{a}	0^{a}	0^{b}	0^{a}
Fanagoria White semi-sweet, 2017	0.130 ±0.028^{b}	7.715 ±0.125^{b}	4.025 ±0.045^{c}	1.161 ±0.088^{b}	3.903 ±0.177^{c}	0^{a}	2.745 ±0.095^{ab}	0.789 ±0.047^{c}	0.77 ±0.03^{c}	0^{a}	0^{a}	0^{a}	0.241 ±0.030^{a}	0^{a}

a) gallic acid, b) caftaric acid, c) coutaric acid, d) caffeic acid, e) fertaric acid, f) myrecitin-3-O-glucoside, g) p-coumaric acid, h) hexose ester of protocatechuic acid, i) dihydroquercetin-3-O-rhamnoside, q) quercetin-3-O-glucoside, k) quercetin-3-O-glucuronide, l) myricetin, m) quercetin, n) kaempferol. ^{a}Means followed by *the same letter* were not different using Student's *t* test; NR - Nomernoy Reserve or Licence Stockpile. The measurement for each wine variety was repeated 3 times, samples were taken from different bottles. $P < 0.05$ was considered statistically significant (for columns).

Table S3. The content of phenolic acids and flavonols in the red Russian Fanogoria wines (mg/L).

Red wine and vintage	a	b	c	d	e	f	g	h	i	q	k	l	m	n
Fanagoria Author's #1, 2017	tr.	74.615 ±0.935	68.825 ±1.605	0.525 ±0.055	9.875 ±0.395	48.635 ±2.825	tr.	0	0	30.513 ±1.663	2.318 ±0.043	8.252 ±0.257	7.571 ±0.360	1.382 ±0.239
Fanagoria Cabernet, 2017	4.615 ±0.275	15.195 ±0.435	10.489 ±0.637	1.655 ±0.125	9.377 ±0.335	40.969 ±3.239	tr.	0	0	11.465 ±0.775	3.475 ±0.275	10.915 ±0.515	6.622 ±0.584	2.705 ±0.115
Fanagoria Cabernet-Saperavi, 2017	3.765 ±0.065	12.670 ±0.055	8.345 ±0.485	1.315 ±0.118	16.415 ±0.185	79.191 ±1.632	tr.	0	0	16.822 ±1.997	5.865 ±0.535	27.645 ±0.855	8.835 ±0.295	3.704 ±0.143
Fanagoria Cahors Canonical, 2016 (1957)	4.921 ±0.054	92.082 ±1.443	8.645 ±0.395	1.395 ±0.155	4.577 ±0.509	43.025 ±1.375	tr.	0	0	4.705 ±0.385	1.785 ±0.135	10.622 ±0.427	4.031 ±0.134	1.964 ±0.186
Fanagoria Cahors Canonical, 2017 (Cahors)	5.685 ±0.145	23.825 ±0.415	20.71 ±2.054	1.675 ±0.525	4.681± 0.047	36.737 ±4.754	tr.	0	0	11.560 ±0.872	3.155 ±0.095	8.175 ±0.385	4.442 ±0.296	2.065 ±0.075
Fanagoria Merlot semi-sweet, 2017	5.368 ±0.093	21.151 ±0.678	14.58 ±0.173	1.848 ±0.056	8.085 ±0.255	52.505 ±1.053	tr.	0	0	17.715 ±0.365	4.845 ±0.065	18.275 ±3.265	12.660 ±0.082	2.235 ±0.195
Fanagoria Merlot, 2016 (NR)	6.419 ±0.295	14.358 ±0.037	8.920 ±0.145	0.633 ±0.048	10.565 ±0.335	51.970 ±0.782	tr.	0	0	16.035 ±0.275	4.329 ±0.243	11.515 ±0.425	10.302 ±0.325	1.997 ±0.129
Fanagoria Merlot, 2016, after six months, storage in dark, +10 °C (NR)	5.983 ±0.571	12.991 ±0.274	8.023 ±0.662	0.604 ±0.136	7.922 ±0.298	40.065 ±1.015	tr.	0	0	7.825 ±0.115	8.954 ±0.036	14.170 ±0.376	4.545 ±0.175	1.901 ±0.298
Fanagoria Merlot (NR), 2017	6.645 ±0.055	14.747 ±0.333	8.13 ±0.567	tr.	15.385 ±0.245	55.435 ±1.385	tr.	0	0	13.131 ±0.161	3.945 ±0.125	17.176 ±0.762	4.158 ±0.272	0.812 ±0.122
Fanagoria Merlot (FS), 2017	4.981 ±0.150	20.582 ±0.538	10.165 ±0.575	tr.	4.445 ±0.065	48.342 ±1.614	tr.	0	0	17.840 ±0.725	4.515 ±0.085	26.115 ±0.595	15.975 ±0.355	4.361 ±0.224
Fanagoria Red semi-sweet, 2017	5.260 ±0.097	25.910 ±0.982	18.145 ±0.465	2.040 ±0.174	4.655 ±0.455	39.310 ±1.082	tr.	0	0	11.775 ±0.225	4.251 ±0.033	16.878 ±1.834	7.072 ±0.039	1.645 ±0.085
Fanagoria Saperavi, 2017	5.855 ±0.215	15.205 ±0.975	6.16 ±3.531	1.103 ±0.042	7.172 ±0.15	54.451 ±5.547	tr.	0	0	11.645 ±0.355	3.332 ±0.225	7.821 ±0.175	6.585 ±0.115	2.431 ±0.023
Fanagoria Saperavi semi-sweet, 2016 (1957)	6.051 ±0.131	14.025 ±0.415	10.721 ±0.546	1.305 ±0.185	7.275 ±0.175	47.461 ±0.685	tr.	0	0	8.183 ±0.377	2.285 ±0.135	5.885 ±0.258	1.811 ±0.177	0.385 ±0.115
Fanagoria Saperavi semi-sweet, 2017 (1957)	6.047 ±0.12	14.541 ±0.453	10.973 ±0.506	1.211 ±0.097	7.573 ±0.304	47.047 ±0.866	tr.	0	0	8.947 ±0.209	2.343 ±0.186	6.473 ±0.293	2.737 ±0.449	0.493 ±0.047
Fanagoria Tsimlyansky black, 2016 (NR)	6.405 ±0.075	17.380 ±0.493	4.225 ±0.035	1.712 ±0.244	12.631 ±0.177	48.551 ±0.295	tr.	0	0	7.825 ±0.215	2.951 ±0.057	14.168 ±1.373	4.545 ±0.145	2.008 ±0.397
Fanagoria Tsimlyansky black, 2017 (NR)	3.831 ±0.128	25.475 ±1.535	5.535 ±0.035	2.125 ±0.505	10.485 ±0.454	65.015 ±0.415	tr.	0	0	11.005 ±0.995	5.210 ±0.996	26.175 ±1.725	4.505 ±0.125	1.831 ±0.215

a) gallic acid, b) caftaric acid, c) coutaric acid, d) caffeic acid, e) fertaric acid, f) myrecitin-3-O-glucoside, g) p-coumaric acid, h) hexose ester of protocatechuic acid, i) dihydroquercetin-3-O-rhamnoside, q) quercetin-3-O-glucoside, k) quercetin-3-O-glucuronide, l) myricetin, m) quercetin, n) kaempferol. The measurement for each wine variety was repeated 3 times, samples were taken from different bottles. $P < 0.05$ was considered statistically significant (for columns); tr. - trace.

Table S4. List of the phenolic compounds from wine extracts

Peak*	Name	Rt (min)	UV, λmax (nm)	Moll mass (g/M)	$(M-H)^-$ (m/z)	MS^2 fragments (m/z)
1	*trans*-piceid	20.6	310	390.388	227	100
2	*trans*-piceatanno	22.6	320	244.246	243	225
3	*cis*-piceid	23.9	290	228.25	227	100
4	*trans*-resveratrol	29.5	310	228.25	227	185
5	*cis*-resveratrol	29.5	290	390.4	227	185
6	δ-viniferin	35.4	320	454.48	453	359,347
a	Gallic acid	7.8	270	170.12	169	125
b	Caftaric acid	11.8	330	312.229	311	149,179
c	Coutaric acid	14	310	296.231	295	163
d	Caffeic acid	15.5	320	180.16	179	135
e	Fertaric acid	15.8	320	326.25	325	193
f	Myrecitin-3-O-glucoside	21.1	360	480.4	479	317,179,151
g	p-Coumaric acid	19.3	310	164.047	163	119
h	Hexose ester of protocatechuic acid	21.1	280	450.4	315	153
i	Dihydroquercetin-3-O-rhamnoside	21.5	360	448.4	449	285,303
j	Quercetin-3-O-glucoside	19.2	360	464.4	463	301
k	Quercetin-3-O-glucuronide	21.2	360	478.1	477	301
l	Myricetin	24.8	360	318.23	317	nd
m	Quercetin	29.1	360	302.236	301	nd
n	Kaempferol	33.2	360	286.23	285	nd

*The peak numbers are shown as in the Fig. 1.
